# Muscle Research: A Tour d’Horizon

**DOI:** 10.3390/ijms23031585

**Published:** 2022-01-29

**Authors:** Carlo Zancanaro

**Affiliations:** Department of Neurological and Movement Sciences, University of Verona, I-37100 Verona, Italy; carlo.zancanaro@univr.it

The Special Issue on the “Muscular Structure, Physiology and Metabolism” was proposed in order to maintain the referenced scientific community abreast with recent research advancements regarding the morphology, functionality, and metabolism of muscle tissue, including a total of eighteen published papers, of which twelve were original research manuscripts and six were review papers.

These papers dealt with diverse aspects of muscle biology, offering an exciting overview of key research myology topics.

Three papers were dedicated to the issue of Ca^2+^ role(s) in muscle cells. The work by Rincon et al. [1] presented a comprehensive mathematical model of the excitation–contraction coupling integrating most Ca^2+^ handling mechanisms in an effort to overcome the limitations of the fast vs. slow fibers dichotomy and the use of slow dyes, expecting its results to provide a better quantitation of store-operated Ca^2+^ entry fluxes and thermal changes in mammalian fiber types, thereby supporting the use of fast Ca^2+^ dyes for most experimental approaches in skeletal muscle. The paper by Romagnoli et al. [2] dealt with the expression and function of the calcium-sensing receptor in human skeletal muscle tissues and satellite cells therefrom. Interestingly, the results suggested that, despite being a very important drug target in the physiology and pathology of other organs, this receptor was not present in healthy human skeletal muscle tissue, derived SCs, and cells differentiating into myoblasts; accordingly, it probably does not have any physiological role in skeletal muscle in normal conditions. The paper from Qaisar et al. [3] explored the pharmacological treatment’s ability to restore the activity of the sarcoplasmic reticulum Ca^2+^ ATPase (SERCA) pump, found to be depressed in sarcopenia. Findings in mice indicated that the pharmacological targeting of SERCA can be an effective therapy to counter age-related muscle dysfunctions, thereby leading the way towards future translational human research.

Three papers in the Special Issue dealt with the redox status in muscle. Fernández-Puente and Palomero [4] showed that genetically encoded biosensors associated with quantitative fluorescence microscopy represent a robust methodology for investigating the pathophysiological processes associated with the redox biology of skeletal muscle. Using mice hearts lacking in cytoplasmic superoxide dismutase, Varshney et al. [5] were able to show a pathological adaptation of the hearts to oxidative stress consisting of an increase in heart weights and concentric hypertrophy. In their review paper, Mirzoev et al. [6] explored the role of glycogen synthase kinase 3β (GSK-3β) in the regulation of protein turnover, myosin phenotype, and oxidative capacity in skeletal muscle under disuse conditions, concluding that GSK-3β may represent a perspective therapeutic target in the treatment of muscle wasting induced by chronic disuse and aging.

Muscle aging was the focus of two papers in the Special Issue. In the first one, Lofaro et al. [7] investigated the proteomics of the skeletal muscle extracellular matrix (matrisome) in aging mice, demonstrating several statistically significantly increased matrisome proteins in old vs. adult animals, where the proteomic findings were confirmed and expanded using morphological data. A second aging paper was presented by Olson et al. [8], showing that deleterious age-dependent collagen modifications were present in a decellularized muscle matrix derived from old mice. The results implied that age is relevant when using a skeletal muscle extracellular matrix as a biomaterial.

The papers by Akberdin et al. [9] and Makhnovskii et al. [10] investigated the effects of physical exercise on muscle tissue. Akberdin et al. [9] developed a physiologically based computational model of skeletal muscle which included an energy metabolism, Ca^2+^, and AMPK (AMP-dependent protein kinase) signaling pathways, as well as the expression regulation of genes with early and delayed responses; Makhnovskii et al. [10] carried out a meta-analysis showing that skeletal muscle adaptation strategies to decrease and increase levels of physical activity differed in direction and demonstrated qualitative differences in association with the activation of different sets of transcription factors.

Aspects of myogenesis were investigated by two papers, one of which was by Da Paixão et al. [11] who confirmed and expanded on palmitic acid negatively affecting myogenesis in vitro, while Chen et al. [12] reviewed the evidence on ion channels and transporters in muscle cell differentiation and concluded that the elucidation of the mechanisms by which ion channels and transporters promoting muscle cell differentiation could help reach a better understanding of muscle development or disease, thereby providing insight for the development of therapeutic strategies.

Leiva-Cepas et al. [13] reported on ultrasonographic and histological correlation after an experimental reconstruction of muscle loss with adipose tissue and found that ultrasound could be a useful tool for evaluating the structure of muscles reconstructed through tissue engineering.

Zanella et al. [14] showed that ascorbic acid supplementation positively influenced muscle growth after fasting in pacu (*Piaractus mesopotamicus*) juveniles.

Three reviews concluded the Special Issue. Romagnoli et al. [15] summarized evidence retrieved from in vitro 2D/3D models of human satellite cells to assess the skeletal muscle biology for pre-clinical investigations; Gomez-Oca et al. [16] presented an exhaustive revision of common pathogenic mechanisms in centronuclear and myotubular myopathies also dealing with latest treatment advances; Aránega et al. [17] presented an extensive review of recent advances highlighting the potential of miRNAs for use in conjunction with gene replacement therapies in order to improve muscle regeneration in the context of Duchenne muscular dystrophy.

Overall, the present Special Issue was very successful and highlighted several interesting aspects of cutting-edge research in myology. As the Guest Editor, I wish to warmly thank all the authors for their significant contributions to this article’s collection, and to thank the *International Journal of Molecular Science* for its support.
